# Alcohol consumption and associated factors among pregnant women attending antenatal care at governmental hospitals in Harari regional state, Eastern, Ethiopia

**DOI:** 10.1186/s13011-023-00567-6

**Published:** 2023-10-31

**Authors:** Tilahun Bete, Henock Asfaw, Kabtamu Nigussie, Addisu Alemu, Addis Eyeberu Gebrie, Deribe Bekele Dechasa, Kabtamu Gemechu, Mesay Arkew, Beniam Daniel, Habtam Gelaye, Asrat Wolde, Mulat Awoke Kassa, Tamrat Anbesaw

**Affiliations:** 1 Department of Psychiatry, School of Nursing and Midwifery, College of Health and Medicine Science, Haramaya University, Harar, Ethiopia; 2 School of Public Health, College of Health and Medical Sciences, Haramaya University, Harar, Ethiopia; 3 Department of Midwifery, School of Nursing and Midwifery, College of Health and Medicine Science, Haramaya University, Harar, Ethiopia; 4 Department of Nursing, School of Nursing and Midwifery, College of Health and Medicine Science, Haramaya University, Harar, Ethiopia; 5 Department of Hematology, School of Medical Laboratory Science, College of Health and Medicine Science, Haramaya University, Harar, Ethiopia; 6 Department of Psychiatry, College of Health and Medicine Science, Arba Minch University, Arba-Minch, Ethiopia; 7 Department of Psychiatry, College of Medicine and Health Science, Wollo University, P.O. Box 1145, Dessie, Ethiopia; 8 Department of Psychiatry, School of Medicine, College of Medicine and Health Sciences, Mizan-Tepi University, Tepi, Ethiopia; 9 Department of Nursing, College of Health Sciences, Woldia University, Woldia, Ethiopia

**Keywords:** Alcohol use, Depression, Pregnant, Harar, Ethiopia

## Abstract

**Background:**

Alcohol consumption during the pregnancy period is high despite the well-established evidence of its harmful effects on pregnancy and infant development. Early identification and behavioral modification are of great significance. This study aimed to assess the prevalence and associated factors of alcohol consumption among pregnant women attending antenatal care at governmental hospitals in the Harari regional state, Eastern Ethiopia.

**Method:**

From April 1/2022-May 1/2022, an institutional-based cross-sectional survey was conducted among 589 pregnant women attending antenatal care governmental hospitals in Harari regional state. A systematic random sampling technique was used to select study participants during the study period. Data were collected through face-to-face interview methods using AUDIT-C. The collected data were coded, entered into Epi-data data version 3.1, and analyzed by SPSS Version 26. Binary logistic regression was carried out to identify independent predictors of alcohol consumption at a 95% confidence level.

**Results:**

From a total of 617 eligible participants, 589 participated in the study with a response rate of 95.46%. The overall prevalence of alcohol consumption among pregnant women in Harari regional state governmental hospitals was 21.2% (95% CI:17.8, 24.4). In multivariate analysis, single marital status (AOR = 5.18;95% CI (2.66,10.11), previous history of abortion(AOR = 4.07;95% CI(2.06,8.04), family history of mental illness (AOR = 4.79;95% CI (1.94,11.83), depression (AOR 2.79; 95%CI(1.35,5.76), and anxiety(AOR = 2.51; 95% CI (1.23, 5.12) were variables found to have a statistically significant association with alcohol consumption during pregnancy in Harari regional state governmental hospitals.

**Conclusion:**

In comparison to the majority of other research, the prevalence of alcohol usage during pregnancy was high in this study**.** This study observed that single marital status, previous history of abortion, family history of mental illness, depression, and anxiety were highly associated with alcohol consumption during pregnancy. Hence, responsible bodies working on mother and child health should try to mitigate or remove the above-mentioned risks when developing interventions.

## Background

Alcohol is the third most preventable risk factor for disease worldwide, accounting for 3.3 million deaths (5.9% of all global deaths) [1]. According to the World Health Organization, alcohol was responsible for 7.6% and 4% of deaths among males and females, respectively. Alcohol is linked to about 200 diseases and injuries, the most common of which are alcoholism, liver cirrhosis, malignancies, and traumas. Alcohol consumption is the 5^th^ greatest cause of premature mortality worldwide, and the first leading cause of death among those aged 15 to 49 [2, 3].

According to the Ethiopian Demographic and Health Survey (EDHS), which included national representative samples from the age group 15–49 years, 53% of men and 45% of women had a lifetime history of alcohol consumption, with 53% of men and 48% of women having consumed alcohol six or more days in the previous month; increasing age and urban residence were associated with high levels of use [4]. In Ethiopia, both manufactured and homemade alcoholic drinks are consumed, with the latter being more popular in rural areas; the estimated alcohol content of various homemade alcoholic drinks was estimated to be 2–4% for tella (traditional beer), 7–11 percent for tej (honey wine), and up to 45 percent for araqe (traditional wine) (strong colorless liquor distilled from grain) [4].

Alcohol consumption during pregnancy is a major public health concern that has adverse health implications for the mother and neonates. Alcohol has harmful consequences on the fetus even if the mother is not dependent There is no amount of alcohol considered to be safe during pregnancy [5]. Alcohol consumption during pregnancy has long been recognized as a risk factor for poor pregnancy outcomes. Stillbirth, spontaneous abortion, early birth, intrauterine growth retardation, and low birth weight are complications of alcohol consumption during pregnancy [6–8].

Fetal alcohol spectrum disorders (FASD) and a variety of other obstetric issues are linked to alcohol usage during pregnancy and the peri-conception period [9, 10]. According to the report of the World Health Organization (WHO), FASD is a serious, yet avoidable, complication of maternal alcohol usage during pregnancy. It includes malformations, memory problems, poor growth, neurodevelopmental delays, poor reasoning and judgment skills, attention deficit hyperactive disorder, and potential behavioral effects on neonates. [11–13]. In addition, prenatal alcohol exposure (PAE) causes lifelong neurocognitive and behavioral impairments [14], which also lead to significant secondary difficulties later in life, including educational failure, substance misuse, mental health problems, and the inability to live independently and to get and maintain a job [15].

Globally, various studies reported that the prevalence of alcohol consumption among pregnant women in Sweden 6% [16], Canada 10% [17], Thailand 5.8% [18], Ghana 20.4 [19], Northern Tanzania 21.5% [20], and Tanzania 15.1% [21], Ghana 48% [22], Ukraine 46.3% [23], Addis Ababa 37.1% [24], Bahir-dar 34% [25], Southern Ethiopia 8.1% [26], and Debre Berhan 16.1% [27]. There are several risks alcohol consumption during the pregnancy period. Some of the risk factors associated with alcohol consumption are being single, urban residence, income, multi-parity, history of mental illness, alcohol consumption before pregnancy, current khat chewing, abortion history, number of children, family history of mental illness, chronic medical illness, depression, anxiety, stress, and not having family social support [16, 17, 20–22, 24–27].

Despite the fact that alcohol drinking during pregnancy has severe physical, emotional, and social consequences, particularly in Ethiopia, few studies have looked into the prevalence and determinants of its use, and there are still few studies to inform programmers and policy-makers. Therefore, this study aimed to assess the prevalence and associated factors of alcohol consumption among pregnant women attending antenatal care at governmental hospitals in the Harari regional state. This study will show the severity to policymakers and different stakeholders to integrate mental health services within the ANC to prevent alcohol consumption. Additionally, it will also serve as baseline data for future studies and researchers.

## Methods and materials

### Study area, period, and study design

An institution-based cross-sectional study was conducted from April 1/2022-May 1/2022 at governmental hospitals in Harari regional state, which is geographically located in Harari regional state is situated 526 km from Addis Ababa in the eastern part of Ethiopia. There are three hospitals. Hiwot Fana Specialized University Hospital, Harari federal police hospital, and Jogol Hospital. The ANC clinic provided services for a total of approximately 1242 pregnant women in the three hospitals. A cross-sectional study design was employed.

### Source population

All pregnant women were attending antenatal care at governmental hospitals in Harari regional state.

### Study population

All pregnant women attending antenatal units at governmental hospitals in Harari regional state who are selected by systematically random sampling technique.

### Inclusion criteria and exclusion criteria

All pregnant women aged 18 and above were included. Whereas those who had difficulty communicating and c critically ill were excluded from the study.

### Sample size determination and sampling technique

#### Sample size estimation

The sample size was determined by using the single population proportion formula. By taking from the proportion of Alcohol consumption in pregnancy 37.1% was taken from another study [24], with a 5% margin of error, 95% CI.

$$\mathrm n=\begin{array}{cc}\frac{(\mathrm Z\mathrm\alpha/2)^2\mathrm P\,(1-\mathrm P)}{\mathrm d^2}=&\frac{\mathrm n\;=\;(1.96)^2\,\mathrm x\,(0.371(1-0.371)=561}{(0.04)^2}\end{array}$$

Therefore, the sample size by adding 5% non-respondent was 617.

### Sampling technique and procedure

The sample was proportionally allocated using the monthly average number of overall pregnant women attending ANC from the registration book for each hospital to make it representative. On average, the total number of pregnant women visiting ANC follow-up is 1242 pregnant women per month. Therefore, as we use the systematic random sampling method, to find the K value, we divide *N* = 1242 to *n* = 617 which becomes ~ 2.01 (K = 2). This was 396 for Hiwot Fana Specialized University Hospital, 29 Harari federal police hospital, and 192 for Jogol Hospital. The study unit (pregnant women) was selected using systematic sampling by determining the sampling interval (K). Therefore, the interval size (K) for each hospital is 2. So, the participants were selected at every 2^nd^ interval. The first pregnant woman was selected from the first three by lottery method who had to follow up during the data collection period_._

### Study variables

#### Dependent variables

Alcohol consumption(Yes /No).

### Independent variables

#### Socio-demographic factors

Age, religion, ethnicity, marital status, educational status, residence, and monthly income.

#### Obstetric, and psychosocial factors

Gravidity, parity, number of children, abortion, alcohol consumption before pregnancy, current khat chewing, and social support.

#### Clinical factors

History of mental illness, family history of mental illness, chronic medical illness (Asthma, HIV/AIDS, hypertension, diabetes, etc.), depression, anxiety, and stress.

#### Data collection method and tools

A structured questionnaire was used to collect the required data, which has different components, including socio-demographic factors, obstetrical factors, clinical factors, psychosocial, and substance-related variables were developed after an extensive review of the literature and similar study tools.

The AUDIT-C is derived from the 10-item alcohol consumption disorder identification test [28, 29]. It was developed by the World Health Organization, which is a cross-culturally validated and preferred tool to be useful in detecting those who have a drinking problem, such as hazardous drinking, dangerous drinking, or alcohol dependency (sensitivity, 94.1%; specificity, 91.7%) [30]. AUDIT-C has been used in a variety of research to assess alcohol consumption in pregnant women [21, 31]. It includes three Likert scale questions that assess the frequency and quantity of alcohol consumption. The AUDIT-C has a total sum score of 12, which can be classified as low risk (1–3), moderate risk (4–5), or high risk (6) based on the total sum score. It includes how often they have a drink containing alcohol, the units of alcohol they drank and how often have they drunk 6 or more units. The AUDIT-C questions were customized for use during pregnancy and contextualized for the study area. Women who had a cumulative score of three or more were classified as alcohol users in this study. Depression, Anxiety, and Stress were assessed using the Depression, Anxiety, and Stress Scale (DASS -21) [32]. Each item contributes 0 to 3 points to the sum score resulting in a total score that intervals from 0 to 21, which is calculated for each scale separately, and the overall score is not calculated. The DASS-21 questionnaire is commonly used in the pregnant population due to the limited number of questions and simple sentences with simultaneous assessment of stress, anxiety, and depression. A score of ten and above was considered as having depression, eight and above having anxiety, and fifteen and above having stress [33]. This instrument is in the public domain and it can also be used in non-clinical populations, and therefore, it can be freely used in research or practice [34]. In the current study, DASS-21 has a high level of internal consistency with Cronbach alphas of 0.81 (depression), 0.84 (anxiety), and 0.78 (stress), for the total scale. The Maternity Social Support Scale (MSSS) was used to measure social support having a maximum sum score of 30. The sum scores were categorized; low social support is defined as a score of less than 18, medium social support is defined as 18–23, and strong social support is defined as 24–30) [35]. The internal consistency (Cronbach alpha) of MSSS in the current study was 0.82. The presence of a known chronic medical condition, such as diabetes, hypertension, or others, as well as family history of mental illness, as reported by self-report (yes/no response).

### Data quality control

Six qualified BSc psychiatric nurses collected data, and one supervisor MSc in psychiatry was also trained to supervise data. Each portion of the questionnaire was written in English, then translated into the local language (Amharic and Afan Oromo) then back-translated into English by an independent person to verify consistency and understandability. The goal of the study, tools, how to collect data, sample strategies, and how to manage ethical problems such as confidentiality were all covered in two days of training for data collectors and a supervisor. Before the main study, a pretest was conducted among 5% of the sample size of pregnant women to detect potential difficulties in the proposed study, such as data collection tools, and to check the performance of the data collectors. Regular supervision of the supervisor and lead investigator was instituted to ensure that all relevant data was obtained in a timely manner. The completed questionnaires were checked for completeness and consistency every day during the data collection period. The data was edited and entered into the computer, then double-checked and processed as needed.

### Data processing and analysis

Data were collected, cleaned, and stored for consistency on a computer using Epi-Data version 3.1, and then exported to SPSS 26 version statistical software for analysis. To choose candidate variables, a bivariate logistic analysis was used. In the multivariate logistic regression model, all variables with a *p*-value of less than 0.25 in the bivariate analysis were included. To compensate for possible confounding effects and determine the presence of a statistically significant link between independent and dependent variables, multivariate logistic regression analysis was used. The appropriate assumptions were checked using Hosmer and Lemeshow goodness. An adjusted odds ratio of 95% CI was used to show the strength of the association, and a *P*-value < 0.05 was considered statistically significant. To describe the study participants about key variables, descriptive statistics findings encompassing frequency, percentages, and summary statistics (mean values and standard deviation) were reported.

## Results

### Socio‑demographic characteristics of participants

A total of 589 participants were included in the study, which resulted in an overall response rate of 95.46%. The mean age (± SD) of the respondents was 27.18 (± 4.14), with an age range of 18–42 years. Of all respondents, the majority were age range of 26–30 years 296 (50.3%). From the participants, 503(85.4%) were married. Nearly half (46.9%) of participants were Muslim in religion followed by orthodox Christian 246(41.8%) and 308(52.3%) were Oromo in their ethnicity. The educational status of participants showed that 242(41.1%) can able to read and write. Large numbers of respondents 410(69.6%) were urban residents. The majority of 509 (86.4%) of the study participants had an average monthly income > 1000 Ethiopian birr (Table 1).

**Table 1:** Socio-demographic characteristics of pregnant women attending antenatal care at governmental hospitals in Harari regional state, Eastern Ethiopia, 2022 (*N* = 589)

Variables	Category	Frequency	Percent (%)
Age	18–25	189	32.1
	26–30	296	50.3
	31–38	104	17.7
Marital status	Single	86	14.6
	Married	503	85.4
Religion	Muslim	276	46.9
	Orthodox	246	41.8
	Protestant	49	8.3
	Others^a^	18	3.1
Ethnicity	Oromo	308	52.3
	Amhara	147	25.0
	Adare	50	8.5
	Gurage	46	7.8
	Others^b^	38	6.4
Level of education	Unable to read and write	65	11.0
	Able to read and write	242	41.1
	Primary school	187	31.7
	Secondary school	73	12.5
	College and above	22	3.7
Residence	Rural	179	30.4
	Urban	410	69.6
Monthly Income( In Ethio Birr)	< 1000	80	13.6
	> 1000	509	86.4

Others: ^a^Adventist & Catholic

^b^Tigrie, Somali & Sidama

### Obstetrics, substance, and psychosocial characteristics of the respondents

From the respondent, 479 (81.32%) and 474(80.5%) were multigravida and multipara respectively. Out of the total participants, 66(11.2%) women had a previous history of abortion, and 64 (15.4%) had abortion intentions in the current pregnancy. The majority of the participants had one child 246 (41.8%). Of the total participants, one-fourth (25.8%) and (27.2%) had pre-pregnancy alcohol consumption history and current khat chewing respectively. Regarding social support, nearly two thirds (61.3%), 152(25.8%), and 76(12.9%) of the pregnant women had received high social support, medium social support, and low social support respectively (Table 2).

**Table 2:** Obstetrics, Substance and Psychosocial characteristics of pregnant women attending antenatal care at governmental hospitals in Harari regional state, Eastern Ethiopia, 2022 (*N* = 589)

Variables	Category	Frequency	Percent (%)
Gravidity	Primigravida	110	18.7
	Multigravida	479	81.3
Parity	Nulliparous	115	19.5
	Multiparous	474	80.5
Abortion	Yes	66	11.2
	No	523	88.8
Number of children	No children yet	115	19.5
	Has one child	246	41.8
	Has two child	155	26.3
	Three and more	73	12.3
Alcohol use before	Yes	152	25.8
Pregnancy	No	437	74.2
Current khat chewing	Yes	160	27.2
	No	429	72.8
Social support	Low social support	76	12.9
	Medium social support	152	25.8
	High social support	361	61.3

### Clinical characteristics of the respondents

According to this study finding, 30(5.1%) of respondents have a previous history of mental illness. Among all participants, 38(6.5%) of respondents have a family history of mental illness. More than one-tenth of participants, 67(11.4%) have a comorbid medical illness, from these medical illnesses, asthma 24(4.1%), HIV/AIDS 20(3.4%), hypertension 12(2.0%), and diabetes 11(1.9%) were accounted. Of the participants, 118(20.0%), 120(20.4%), and 244(41.4%) have depression, anxiety, and stress symptoms respectively (Table 3).

**Table 3:** Clinical characteristics of pregnant women attending antenatal care at governmental hospitals in Harari regional state, Eastern Ethiopia, 2022 (*N***=**589)

Variables	Category	Frequency	Percent (%)
Past mental illness history	Yes	30	5.1
	No	559	94.9
Family history of mental illness	Yes	38	6.5
	No	551	93.5
Chronic medical illness	Yes	67	11.4
	No	522	88.6
Asthma	Yes	24	4.1
	No	565	95.9
HIV/AIDS	Yes	20	3.4
	No	569	96.6
Hypertension	Yes	12	2.0
	No	578	98.0
Diabetes	Yes	11	1.9
	No	578	98.1
Depression	Yes	118	20.0
	No	471	80.0
Anxiety	Yes	120	20.4
	No	469	79.6
Stress	Yes	244	41.4
	No	345	58.6

### The magnitude of alcohol consumption among pregnant women attending antenatal care at governmental hospitals in Harari regional state, Eastern Ethiopia

In the present study, the prevalence of alcohol consumption among pregnant women in Harari regional state governmental hospitals was 21.2% (95% CI:17.8, 24.4).

### Factors associated with alcohol consumption among pregnant women

In the bivariate analysis, marital status, residence, income, abortion history, family history of mental illness, chronic medical illness, depression, anxiety, and social support showed a *p*-value of < 0.25 and became a candidate for multivariate analysis. In multivariate binary logistic regression variables; marital status, abortion history, family history of mental illness, depression, and anxiety were found to be statistically associated with alcohol consumption at a *p*-value less than 0.05.

The odds of alcohol consumption among participants with a single marital status were 5.18 times higher as compared to married women [AOR = 5.18;95% CI (2.66,10.11)]. Those pregnant women who had a previous history of abortion were 4.07 times more likely to have alcohol consumption as compared with respondents who did not have a history of abortion [AOR = 4.07;95% CI(2.06,8.04)]. Those pregnant women with a family history of mental illness were about 4.79 times more likely to have alcohol consumption than their counterparts [AOR = 4.79;95% CI (1.94,11.83)]. Regarding depression, the participants with depression were about 2.79 times more likely to use alcohol than their counterparts [AOR 2.79; 95%CI (1.35,5.76)]. Likewise, participants with anxiety were 2.51 times more likely to use alcohol as compared with women who do not use alcohol [AOR = 2.51; 95% CI (1.23, 5.12)] Table 4).

**Table 4:** Bivariate and multivariate logistic regression analysis results of alcohol consumption among pregnant women attending antenatal care at governmental hospitals in Harari regional state, Eastern Ethiopia, 2022 (*N* = 589)

Variables	Category	Alcohol consumption		COR(95%C.I)	AOR(95%C.I)	*P*-values
		**Yes**	**No**			
Marital status	Single	58 (67.4%)	28 (32.6%)	13.48 (8.02,22.65)	5.18 (2.66,10.11)	< 0.0001*
	Married	67 (13.3%)	436 (86.7%)	1	1	
Residence	Urban	97 (23.7%)	313 (76.3%)	1.67 (1.05,2.65)	1.68 (0.93,3.06)	0.087
	Rular	28 (15.6%)	151 (84.4%)	1	1	
Income	< 1000	29 (36.3%)	51 (63.8%)	2.44 (1.47,4.06)	1.05 (0.49,2.24)	0.889
	> 1000	96 (18.9%)	413 (81.1%)	1	1	
Abortion	Yes	39 (59.1%)	27 (40.9%)	7.34 4.26,12.63)	4.07 (2.06,8.04)	< 0.0001*
	No	86 (16.4%)	437 83.6%)	1	1	
Family history of mental illness	Yes	25 65.8%	13 34.2%)	8.67 (4.29,17.54)	4.79 1.94,11.83)	0.001*
	No	100 18.1%)	451 81.9%)	1	1	
Chronic medical illness	Yes	24 35.8%)	43 64.2%)	2.33 1.35,4.01)	0.58 0.26,1.30)	0.187
	No	101 19.3%)	421 80.7%)	1	1	
Depression	Yes	72 61.0%)	46 39.0%)	12.34 7.73,19.70)	2.79 1.35,5.76)	0.005*
	No	53 11.3%)	418 88.7%)	1	1	
Anxiety	Yes	66 55.0%)	54 45.0%)	8.49 5.41,13.34)	2.51 1.23,5.12)	0.011*
	No	59 12.6%)	410 87.4%)	1	1	
Social support	Poor	29 38.2%)	47 61.8%)	3.03 1.77,5.20)	1.28 0.59,2.76)	0.531
	Moderate	35 23.0%)	117 77.0%)	1.47 0.92,2.35)	1.84 0.99,3.43)	0.054
	Strong	61 16.9%)	300 83.1%)	1	1	

*COR* Crude Odds Ratio, *AOR* Adjusted odds Ratio, 1 = reference category, Hosmer Lemeshow goodness-of-fit 0.83, degrees of freedom = 8, Maximum VIF = 2.3

^*^Statistically significant at *P*-value < 0.05

## Discussion

This study aimed to assess the prevalence of alcohol consumption during pregnancy and associated factors attending antenatal care services. The findings revealed that about 21.2% (95% CI:17.8, 24.4) of women use alcohol during pregnancy and this use was significantly associated with marital status, abortion history, family history of mental illness, depression, and anxiety. This result was in line with other findings done in Ghana 20.4% [19], and Northern Tanzania 21.5% [20]. On the other hand, this study finding was higher when compared with a study done in Southern Ethiopia 8.1% [26], Sweden 6% [16], Canada 10% [17], Thailand 5.8% [18], Debre Berhan 16.1% [27], and Tanzania 15.1% [21]. The discrepancy might be due to only 18 weeks of gestational age and above pregnant women being included in the study in Sweden, which lowers the magnitude of alcohol consumption, especially for those women who didn’t aware their pregnancy at the beginning of pregnancy and they may drink alcohol [16]. Another reason for the disparity could be the tool employed; in Thailand and Sweden, alcohol consumption was measured using ASSIST-lite and AUDIT-C, respectively.

However, in some other studies, the proportion of alcohol consumption was higher than in the current study, a study conducted in Ghana 48% [22], Ukraine 46.3% [23], Addis Ababa 37.1% [24], Bahir-dar 34% [25]. The disparity could be due to a difference in study design, such as a prospective cohort study done in two regions of Ukraine [23]. Also, the study setting where samples were collected was another possible variation, a community-based study was conducted among women in James Town, Ghana [22]. The lower magnitude in the present study might be due to, cultural and religious influence on the study participants, in this study, the majority of the study participants were 276 (46.9%) Muslims in their religion and their doctrine of religion prevents them to use alcohol. Another disparity could be explained by differences in the screening tools utilized. Additionally, the study in Bahir-Dar utilized T-ACE to screen for alcohol consumption, which is more sensitive than AUDIT-C [36]. Furthermore, a study done in Bahir-Dar [25] was a community-based that might benefit all pregnant women. However, only pregnant women who had antenatal care follow-up were included in our study that may raise the possibility of underreporting consumption because the questions were asked while the women are still pregnant. They may deny or minimize consumption because they may be afraid of consequences, being blamed if the child has a problem, and taken away.

Regarding the associated factors, in this study, the odds of using alcohol were more than five times more occur among a single marital status than married participants. Lack of cohabiting partners is more likely to have similar drinking and other substance usage tendencies. Having no one to talk to and a lack of social support can lead to people drinking alcohol more to cope with stress and make depression worse [37]. Furthermore, a lack of emotional support, such as encouragement to use, can play a significant role in continuing to drink [24].

In the current study, we found that women who had a history of abortion were 4.07 times more likely to have alcohol consumption as compared with respondents who do not have a history of abortion. These findings are congruent with another study carried out in Southern Ethiopia [26]. This could be due to the fact that women who have experienced pregnancy-related difficulties have a higher incidence of substance use and other mental health issues such as depression as well as anxiety [38]. A Tanzanian study also found that pregnant mothers who have, past history of pregnancy complications act as a protective factor against alcohol usage during pregnancy [21]. This might be attributed to the fact that some women may identify previous pregnancy complications with the unfavorable effects of alcohol, which could lead them to abstain from drinking during their next pregnancy. On the other hand, others may experience psychological discomfort as a result of a pregnancy-related issue, leading them to turn to alcohol even if they also didn’t know how harmful it can be for the fetus to cope with the stress.

Those pregnant women with a family history of mental illness were about 4.79 times more likely to have alcohol consumption than their counterparts. A current study finding was congruent with a finding from Debre Berhan [27]. This might be due to individuals who have been diagnosed with a family history of psychiatric disorders being more likely to have poor mental health and may use alcohol to cope with stress.

We also found that women who had depression were nearly 3 times more likely to use alcohol as compared to their counterparts. This finding was in line with another previous study from Debere Birhan [27], a systematic review and meta-analysis done in Sub-Saharan Africa [39], which showed that depression was significantly associated with women with substance use problems. According to the findings of some studies, various psychiatric symptoms such as depression and alcohol intake can co-occur. This could be because some women use alcohol as a self-medication to cope with sadness [40, 41], or depression can also occur as a result of excessive alcohol consumption [42]. Finally, pregnant women with anxiety were 2.51 times more likely to risk use alcohol compared with those women who had no anxiety. This matches research done from Debere Birhan [27] and Australia [43]. The link could be related to the fact that participants with poor mental health are more likely to self-medicate with alcohol [40, 41].

### Limitations

Biomarker testing for alcohol usage was not evaluated and only self-report was used to ascertain alcohol consumption in this study. Because determining the amount of alcohol in local drinks by volume and ounces is challenging, the number of standard drinks drunk by pregnant women per day was not analyzed. Another limitation was the use of the AUDIT-C and asking women during their pregnancy instead of before or after birth (when they are more likely to admit consumption. The volume and frequency of alcohol usage during pregnancy may be underestimated due to self-reporting, which is prone to social desirability and recollection bias. Because of the cross-sectional character of the study, the cause-and-effect correlations between alcohol consumption and other variables may be missed.

## Conclusion

In comparison to the majority of other research, the prevalence of alcohol consumption during pregnancy was high in the current study. Single marital status, previous history of abortion, family history of mental illness, depression, and anxiety were variables that are independent predictors of alcohol consumption during pregnancy. Alcohol consumption screenings and problematic alcohol consumption evaluations for diagnosis of problems early intervention should be carried out. In addition to this woman before becoming pregnant it is better to reduce consumption and better plan her pregnancy. The effectiveness of brief interventions is higher before pregnancy than during.

## Data Availability

All data generated or analyzed during this study are included in this published article. The data sets of the current study are available from [Tamrat Anbesaw, email: tamratanbesaw@gmail.com; Mobile: + 251(0)9–11289143, Wollo University, Dessie upon reasonable request.

## Abbreviations

AIDS: Acquired Immune Deficiency Syndrome
ANC: Antenatal Care
AOR: Adjusted odds Ratio
ASSIST: Alcohol, Smoking, and Substance Involvement Screening Test
AUDIT-C: Alcohol Use Disorders Identification Test for Consumption
CI: Confidence Interval
COR: Crude Odds Ratio
DASS: Depression, Anxiety, and Stress scale
EDHS: Ethiopia Demographic and Health Survey
FAS: Fetal Alcohol Syndrome
MSSS: Maternal social support scale
SPSS: Statistical Package for Social Science;
WHO: World Health Organization

## References

[1] Asrani SK, Devarbhavi H, Eaton J, Kamath PS (2019). Burden of liver diseases in the world. J Hepatol.

[2] Lim SS, Vos T, Flaxman AD, Danaei G, Shibuya K, Adair-Rohani H (2012). A comparative risk assessment of burden of disease and injury attributable to 67 risk factors and risk factor clusters in 21 regions, 1990–2010: a systematic analysis for the Global Burden of Disease Study 2010. Lancet.

[3] World Health Organization. Global status report on alcohol and health 2018. World Health Organization; 2018. Available at: https://apps.who.int/iris/handle/10665/274603. License: CC BY-NC-SA 3.0 IGO.

[4] CSA-Ethiopia I. International: Ethiopia demographic and health survey 2011. Central Statistical Agency of Ethiopia and ICF International Addis Ababa, Ethiopia and Calverton, Maryland, USA. 2012.

[5] Crawford-Williams F, Steen M, Esterman A, Fielder A, Mikocka-Walus A (2015). “If you can have one glass of wine now and then, why are you denying that to a woman with no evidence”: knowledge and practices of health professionals concerning alcohol consumption during pregnancy. Women Birth.

[6] Kinney J, Leaton G (2000). Loosening the grip: A handbook of alcohol information.

[7] Kesmodel U, Wisborg K, Olsen SF, Henriksen TB, Secher NJ (2002). Moderate alcohol intake during pregnancy and the risk of stillbirth and death in the first year of life. Am J Epidemiol.

[8] Henriksen TB, Hjollund NH, Jensen TK, Bonde JP, Andersson A-M, Kolstad H (2004). Alcohol consumption at the time of conception and spontaneous abortion. Am J Epidemiol.

[9] Williams JF, Smith VC, Levy S, Ammerman SD, Gonzalez PK, Ryan SA (2015). Fetal alcohol spectrum disorders. Pediatrics.

[10] Nykjaer C, Alwan NA, Greenwood DC, Simpson NA, Hay AW, White KL (2014). Maternal alcohol intake prior to and during pregnancy and risk of adverse birth outcomes: evidence from a British cohort. J Epidemiol Community Health.

[11] Sanou AS, Diallo AH, Holding P, Nankabirwa V, Engebretsen IMS, Ndeezi G (2017). Maternal alcohol consumption during pregnancy and child’s cognitive performance at 6–8 years of age in rural Burkina Faso: an observational study. PeerJ.

[12] Popova S, Lange S, Shield K, Mihic A, Chudley AE, Mukherjee RA (2016). Comorbidity of fetal alcohol spectrum disorder: a systematic review and meta-analysis. Lancet.

[13] Sbrana M, Grandi C, Brazan M, Junquera N, Nascimento MS, Barbieri MA (2016). Alcohol consumption during pregnancy and perinatal results: a cohort study. Sao Paulo Med J.

[14] Popova S, Lange S, Poznyak V, Chudley AE, Shield KD, Reynolds JN (2019). Population-based prevalence of fetal alcohol spectrum disorder in Canada. BMC Public Health.

[15] Streissguth AP, Barr HM, Kogan J, Bookstein FL. Understanding the occurrence of secondary disabilities in clients with fetal alcohol syndrome (FAS) and fetal alcohol effects (FAE). Final report to the Centers for Disease Control and Prevention (CDC). 1996. p. 96–06.

[16] Skagerström J, Alehagen S, Häggström-Nordin E, Årestedt K, Nilsen P (2013). Prevalence of alcohol use before and during pregnancy and predictors of drinking during pregnancy: a cross sectional study in Sweden. BMC Public Health.

[17] Lange S, Quere M, Shield K, Rehm J, Popova S (2016). Alcohol use and self-perceived mental health status among pregnant and breastfeeding women in Canada: a secondary data analysis. BJOG.

[18] Assanangkornchai S, Saingam D, Apakupakul N, Edwards JG (2017). Alcohol consumption, smoking, and drug use in pregnancy: prevalence and risk factors in Southern Thailand. Asia Pac Psychiatry.

[19] Adusi-Poku Y, Edusei AK, Bonney AA, Tagbor H, Nakua E, Otupiri E (2012). Pregnant women and alcohol use in the Bosomtwe district of the Ashanti region-Ghana. Afr J Reprod Health.

[20] Isaksen AB, Østbye T, Mmbaga BT, Daltveit AK (2015). Alcohol consumption among pregnant women in Northern Tanzania 2000–2010: a registry-based study. BMC Pregnancy Childbirth.

[21] Mpelo M, Kibusi SM, Moshi F, Nyundo A, Ntwenya JE, Mpondo BC (2018). Prevalence and factors influencing alcohol use in pregnancy among women attending antenatal care in Dodoma region, Tanzania: a cross-sectional study. J Pregnancy.

[22] Da Pilma LJ, Dako-Gyeke P, Agyemang SA, Aikins M (2017). Alcohol consumption among pregnant women in James town community, Accra Ghana. Reprod Health.

[23] Chambers CD, Yevtushok L, Zymak-Zakutnya N, Korzhynskyy Y, Ostapchuk L, Akhmedzhanova D (2014). Prevalence and predictors of maternal alcohol consumption in 2 regions of Ukraine. Alcohol Clin Exp Res.

[24] Tesfaye G, Demlew D, Habte F, Molla G, Kifle Y, Gebreegziabhier G (2020). The prevalence and associated factors of alcohol use among pregnant women attending antenatal care at public hospitals Addis Ababa, Ethiopia, 2019. BMC Psychiatry.

[25] Anteab K, Demtsu B, Megra M (2014). Assessment of prevalence and associated factors of alcohol use during pregnancy among the dwellers of Bahir-Dar City, Northwest Ethiopia, 2014. Int J Pharma Sci Res.

[26] Mekuriaw B, Belayneh Z, Shemelise T, Hussen R (2019). Alcohol use and associated factors among women attending antenatal care in Southern Ethiopia: a facility based cross sectional study. BMC Res Notes.

[27] Wubetu AD, Habte S, Dagne K (2019). Prevalence of risky alcohol use behavior and associated factors in pregnant antenatal care attendees in Debre Berhan, Ethiopia, 2018. BMC Psychiatry.

[28] López MB, Lichtenberger A, Conde K, Cremonte M (2017). Psychometric properties of brief screening tests for alcohol use disorders during pregnancy in Argentina. Rev Bras Ginecol Obstet.

[29] Bradley KA, Bush KR, Epler AJ, Dobie DJ, Davis TM, Sporleder JL (2003). Two brief alcohol-screening tests From the Alcohol Use Disorders Identification Test (AUDIT): validation in a female Veterans Affairs patient population. Arch Intern Med.

[30] Saunders JB, Aasland OG, Babor TF, De la Fuente JR, Grant M (1993). Development of the alcohol use disorders identification test (AUDIT): WHO collaborative project on early detection of persons with harmful alcohol consumption-II. Addiction.

[31] Lee SH, Shin SJ, Won S-D, Kim E-J, Oh D-Y (2010). Alcohol use during pregnancy and related risk factors in Korea. Psychiatry Investig.

[32] Henry JD, Crawford JR (2005). The short-form version of the Depression Anxiety Stress Scales (DASS-21): construct validity and normative data in a large non-clinical sample. Br J Clin Psychol.

[33] Anbesaw T, Negash A, Mamaru A, Abebe H, Belete A, Ayano G (2021). Suicidal ideation and associated factors among pregnant women attending antenatal care in Jimma medical center, Ethiopia. PLoS One.

[34] Lee E-H, Moon SH, Cho MS, Park ES, Kim SY, Han JS (2019). The 21-item and 12-item versions of the Depression Anxiety Stress Scales: Psychometric evaluation in a Korean population. Asian Nurs Res.

[35] Anbesaw T, Abebe H, Kassaw C, Bete T, Molla A (2021). Sleep quality and associated factors among pregnant women attending antenatal care at Jimma Medical Center, Jimma, Southwest Ethiopia, 2020: cross-sectional study. BMC Psychiatry.

[36] Smith L, Savory J, Couves J, Burns E (2014). Alcohol consumption during pregnancy: cross-sectional survey. Midwifery.

[37] Moon TJ, Mathias CW, Mullen J, Karns-Wright TE, Hill-Kapturczak N, Roache JD (2019). The role of social support in motivating reductions in alcohol use: a test of three models of social support in alcohol-impaired drivers. Alcohol Clin Exp Res.

[38] Coleman PK, Coyle CT, Shuping M, Rue VM (2009). Induced abortion and anxiety, mood, and substance abuse disorders: isolating the effects of abortion in the national comorbidity survey. J Psychiatr Res.

[39] Addila AE, Bisetegn TA, Gete YK, Mengistu MY, Beyene GM (2020). Alcohol consumption and its associated factors among pregnant women in Sub-Saharan Africa: a systematic review and meta-analysis' as given in the submission system. Subst Abuse Treat Prev Policy.

[40] Zhong B-L, Xu Y-M, Xie W-X, Lu J, Yu W-B, Yan J (2019). Alcohol drinking in Chinese methadone-maintained clients: a self-medication for depression and anxiety?. J Addict Med.

[41] Gould LF, Hussong AM, Hersh MA (2012). Emotional distress may increase risk for self-medication and lower risk for mood-related drinking consequences in adolescents. Int J Emot Educ.

[42] Subramaniam M, Mahesh MV, Peh CX, Tan J, Fauziana R, Satghare P (2017). Hazardous alcohol use among patients with schizophrenia and depression. Alcohol.

[43] Giglia R, Binns C (2007). Patterns of alcohol intake of pregnant and lactating women in Perth Australia. Drug Alcohol Rev.

